# Wnt signaling regulates chemokine production and cell migration of circulating human monocytes

**DOI:** 10.1186/s12964-024-01608-8

**Published:** 2024-04-16

**Authors:** Natalie Zelikson, Shaina Ben, Michal Caspi, Raneen Tarabe, Yonatan Shaleve, Yael Pri-Paz Basson, Oshrat Tayer-Shifman, Elad Goldberg, Shaye Kivity, Rina Rosin-Arbesfeld

**Affiliations:** 1 Department of Clinical Microbiology and Immunology, Faculty of Medical & Health Sciences, Tel Aviv University, Tel Aviv, Israel; 2 Rheumatology Unit, Meir Medical Center, Kfar Saba, Israel; 3 Department of Medicine F, Rabin Medical Center, Beilinson Hospital, Petah Tikva, Israel; 4 Faculty of Medical & Health Sciences, Tel Aviv University, Tel Aviv, Israel

## Abstract

The β-catenin dependent canonical Wnt signaling pathway plays a crucial role in maintaining normal homeostasis. However, when dysregulated, Wnt signaling is closely associated with various pathological conditions, including inflammation and different types of cancer.

Here, we show a new connection between the leukocyte inflammatory response and the Wnt signaling pathway. Specifically, we demonstrate that circulating human primary monocytes express distinct Wnt signaling components and are susceptible to stimulation by the classical Wnt ligand—Wnt-3a. Although this stimulation increased the levels of β-catenin protein, the expression of the classical Wnt-target genes was not affected. Intriguingly, treating circulating human monocytes with Wnt-3a induces the secretion of cytokines and chemokines, enhancing monocyte migration. Mechanistically, the enhanced monocyte migration in response to Wnt stimuli is mediated through CCL2, a strong monocyte-chemoattractant.

To further explore the physiological relevance of these findings, we conducted *ex-vivo* experiments using blood samples of patients with rheumatic joint diseases (RJD) – conditions where monocytes are known to be dysfunctional. Wnt-3a generated a unique cytokine expression profile, which was significantly distinct from that observed in monocytes obtained from healthy donors.

Thus, our results provide the first evidence that Wnt-3a may serve as a potent stimulator of monocyte-driven immune processes. These findings contribute to our understanding of inflammatory diseases and, more importantly, shed light on the role of a core signaling pathway in the circulation.

**Supplementary Information:**

The online version contains supplementary material available at 10.1186/s12964-024-01608-8.

## Introduction

Secreted Wnt glycoproteins regulate numerous biological processes by initiating a variety of signaling cascades. The canonical Wnt pathway is transduced through the transcription factor β-catenin, leading to the expression of genes involved in cell fate, differentiation, and proliferation [1–4]. The pathway is initiated by Wnt ligand binding to specific receptors and co-receptors from the Frizzled (FZD) family and the low-density lipoprotein receptor-related family, LRP5 or LRP6. This interaction leads to membrane recruitment and oligomerization of Disheveled (DVL), inhibiting a cytoplasmic complex dedicated to phosphorylation and degradation of β-catenin, the core component of the canonical Wnt pathway. This promotes the accumulation and nuclear translocation of β-catenin, leading to the upregulation of Wnt target genes [2, 5, 6]. Unregulated activation of the β-catenin-dependent Wnt pathway is a key factor in the development and progression of many tumor types, most notably in colorectal cancer [2, 7]. However, emerging data now demonstrate that this signal transduction pathway is also involved in other solid and hematological malignancies [8]. Aberrant canonical Wnt signaling is also implicated in the development and progression of inflammation processes, including immunological responses in the lung, intestine, and other systemic diseases, possibly through recently discovered cross-talk between the Wnt and NF-κB pathways [9]. In addition, Wnt signaling has also been shown to affect the regulation of immune cells [10]. Yet, Wnt signaling in circulating white blood cells (WBCs) remains not well-defined, as the complex functions of the Wnt cascade are mostly cell-type- and tissue-specific [11], and have usually been studied in the context of adherent cells and tissues.

Circulating WBCs consist largely of polymorphonuclear cells (neutrophils, eosinophils, and basophils) and peripheral blood mononuclear cells (PBMCs; lymphocytes and monocytes). Monocytes, which arise in the bone marrow and differentiate into macrophages or dendritic cells following organ infiltration, are known to be an important source of Wnt ligands [12, 13]. In the current study, we examined the effect of the classical canonical Wnt ligand, Wnt-3a, [14] that has been shown to promote self-renewal of hematopoietic stem cells [15]. We used primary classical monocytes (CD14^+^CD16^−^, henceforth: “monocytes”) [16], which account for approximately 92% of all human monocytes [17]. Monocytes secrete a variety of proteins, including pro-inflammatory cytokines such as IL-1β [18], anti-inflammatory cytokines such as TGFβ [19] and chemoattractants for various cell types, such as CXCL8 for neutrophils [20], or CCL5 that attracts both T-lymphocytes and monocytes [21] as well as the non-canonical Wnt-5a ligand [22]. Consequently, monocytes play important roles in immune defense, inflammation, tissue remodeling, and self-recruitment/movement to sites of inflammation, which are all critical for the host defense [23]. Monocyte migration depends on numerous chemokines, where the most characterized chemoattractant is CCL2 (also known as MCP-1) [24]. CCL3 (MIP-1α), CCL4 (MIP-1β), the aforementioned CCL5 (RANTES) and CCL7 (MCP-3) are also important factors that influence monocyte function and movement [24]. CCL2 is a member of the chemokine family, which are small, secreted, chemotactic cytokines, named after their best-known function of attracting cells [25]. The protein is expressed by a large number of different cells and its expression is induced by numerous inflammatory stimuli [26]. CCL2 interacts with and activates the seven-transmembrane G-protein-coupled receptor C–C chemokine receptor type 2 (CCR2) [27] to trigger intracellular downstream signaling cascades that are involved in promoting cell migration [28]. Monocytes can switch between pro-inflammatory and anti-inflammatory phenotypes and may act like a double-edged sword in contributing to the pathogenesis of a variety of disorders [29]. For example, rheumatic joint diseases (RJD) involve monocytes and macrophages that contribute to disease pathogenesis (reviewed in [30, 31]). A number of studies using different systems have demonstrated that canonical Wnt signaling can induce an anti-inflammatory cytokine signature in macrophages, which results in a reduction in TNFα [32] and IL6 [33] and an increase in IL4 [34]. However, the role of Wnt signaling in circulating monocytes is not clear.

The results of this study demonstrate that monocytes respond to a Wnt-3a stimulus by an increase in β-catenin levels and changes in the secretion profile of various cytokines and chemokines, as well as changes in migration rates. A notable change was observed in the secretion and reaction to CCL2 and CCL7, which are known to bind CCR2 and guide monocytes to exit the bone marrow and move through the bloodstream toward sites of inflammation [24].

Our results illuminate a yet unknown Wnt signaling cascade in circulating human monocytes, where CCL2, a well-established monocyte-chemoattractant, may serve as a key downstream effector in Wnt-induced monocyte migration. Importantly, our results suggest that canonical Wnt signaling can induce the secretion of a specific set of chemokines and cytokines from circulating monocytes of RJD patients, pointing to a key role of Wnt signaling in this family of diseases.

## Results

### Human monocytes express Wnt signaling components, and the Wnt-3a ligand is found in human plasma

**Fig. 1:**
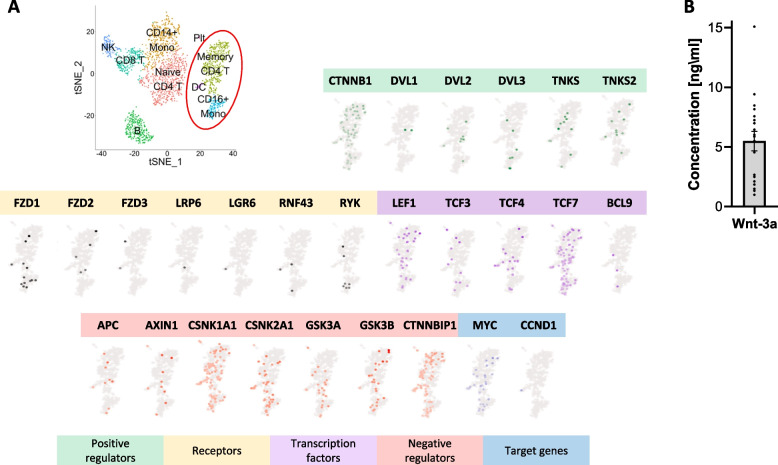
Human monocytes express Wnt signaling components and the Wnt-3a ligand is found in the plasma. **A** Analysis of single-cell RNA-seq data by 10X genomics, based on a Seurat tutorial (“pbmc3k”). tSNE plot of the data with annotation of cell types (upper left panel). Classical monocytes are the orange cluster labeled “CD14^+^ Mono” and non-classical monocytes are the light blue cluster labeled “CD16^+^ Mono”. Plt – platelets. NK – natural killer cells. B and T lymphocytes are abbreviated B and T, respectively. The monocyte cluster (red circle) was further analyzed for expression of Wnt signaling components. The genes are grouped by functional categories (color-coded at the bottom). **B** Wnt-3a levels were measured by ELISA in isolated human plasma obtained from multiple healthy donors. Protein concentrations were calculated by regression from a standard curve

To transduce an active canonical Wnt signal, the cell must express both membrane and intracellular Wnt signaling components. In this context, previous studies have demonstrated the expression of FZD receptors and TCF nuclear factors in circulating monocytes [35, 36]. Here, we used single-cell RNA sequencing data obtained from two independent sources (see Material and Methods section) to examine the transcription of multiple Wnt signaling components in different PBMC sub-populations. Figure 1A presents a tSNE plot of the data, annotating cell types. Feature plots of the expression of Wnt signaling-related genes, cropped for classical (CD14^+^CD16^−^ Mono, the focus of this study) and non-classical (CD16^+^ Mono) monocytes, are also shown. Positive and negative regulators of the canonical Wnt pathway have been detected in monocytes, as well as receptors, transcription factors and classical target genes (Fig. 1A, with the uncropped PBMC panel shown in Fig. S1). Similar results were also found in data obtained from another data source (Fig. S2). Wnt signaling is initiated following ligand-receptor binding. Thus, we examined the expression of Wnt-3a, one of the major canonical Wnt signaling ligands, in human plasma. Our results show that Wnt-3a can be detected in the plasma, in concentrations ranging from 1–10 ng/ml (Fig. 1B). These amounts are sufficient for receptor-ligand activation in the circulation as cytokine concentrations are commonly measured in pg/ml [37], and the levels are similar to the previous levels of Wnt-5a reported in human plasma [22]. The findings of a functional concentration of the canonical Wnt signaling ligand in human plasma, and the expression of different pathway components in circulating monocytes, suggest that canonical Wnt signaling may be active in monocytes.

### Wnt-3a induces expression and nuclear accumulation of β-catenin in monocytes

**Fig. 2:**
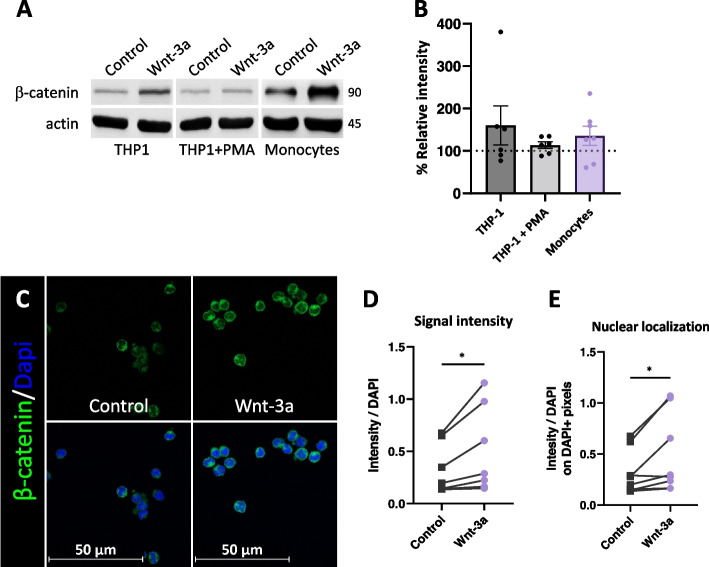
Wnt-3a induces expression and nuclear accumulation of β-catenin in monocytes. **A** and** B**, THP-1 cells (monocyte-like cells), PMA-treated THP-1 cells (macrophage-like cells), and freshly isolated primary monocytes were cultured for 5 h in the presence of control or Wnt-3a conditioned media (CM). **A** Cells were lysed, and western blot analysis was conducted using specific antibodies as indicated (representative blot). **B** Quantification of band intensity of experiments as in A, calculated by the ImageJ software. The Y-axis represents the band intensity of β-catenin standardized to actin (β-catenin/actin) of Wnt-treated cells relative to control-treated cells (100%). **C-E** Treated monocytes (as in A) were stained and visualized using immunofluorescence. Images were obtained using a confocal microscope with a 60X oil-immersion objective lens **C**. An independent script was used to quantify the signal intensity and nuclear localization of β-catenin. Pairs signify individual donors, where each point represents the mean value of at least 5 fields **D, E** (see methods section). *P-value = 0.0359 for paired t-test of signal intensity **D** and *P-value = 0.0342 for paired t-test of nuclear localization **E**

Canonical Wnt signaling is β-catenin-dependent [4]. To assess the potential of activating canonical Wnt signaling we used conditioned media (CM) prepared from commercially available L-Wnt-3a cells, which are mouse fibroblasts stably expressing the human Wnt-3a protein. CM derived from L-cells, containing the same proteins with the exception of Wnt-3a, were used as control media. We used the monocyte-like cell line (THP-1), with and without phorbol-12-myristate-13-acetate (PMA) treatment, as a model for monocytes and macrophages, respectively [38]. THP-1 cells have previously been shown to express low levels of β-catenin [35], and treating these cells with Wnt-3a led to a moderate increase in β-catenin expression in the monocytic cell line, compared to the PMA-differentiated macrophages, where no change in β-catenin levels was detected (Fig. 2A, B). A similar increase in β-catenin expression was seen in monocytes isolated from freshly donated human blood. Moreover, immunofluorescence staining revealed β-catenin upregulation and accumulation of nuclear β-catenin following Wnt-3a treatment (Fig. 2C-E).

### Wnt-3a does not induce expression of classical Wnt signaling target genes in monocytes

**Fig. 3:**
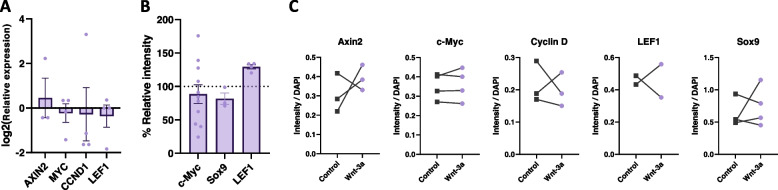
Wnt-3a does not induce expression of classical Wnt signaling target genes in monocytes. **A** Treated monocytes (as in Fig. 2) were lysed for RT-qPCR analysis of the indicated Wnt target genes. **B** Cell protein lysates were analyzed by western blot using specific antibodies as indicated. The graphs represent the relative band intensity. **C** Treated monocytes were stained and visualized using immunofluorescence with the indicated antibodies. Signal intensity was quantified with no significant differences for any paired t-test

Protein analysis of THP-1 cells and primary monocytes revealed that both cell types express core canonical Wnt signaling components, including the LRP5/6 receptors, TCF4 (also shown in [35]), GSK3 and the classical Wnt target genes: c-Myc and LEF1 (Fig. S3). Nevertheless, we could not detect increased expression of canonical Wnt target genes in Wnt-3a-treated primary monocytes (Fig. 3). The inability of Wnt-3a to upregulate these common canonical targets was observed at both the RNA level (Fig. 3A) and the protein level (Fig. 3B, C). Our results are supported by previous reports showing that activation of canonical Wnt signaling in THP-1 and monocytes in other experimental systems does not lead to the expression of these classical Wnt target genes, which were mainly characterized in epithelial colon cancer cells [35, 36]. Although recruitment of nuclear β-catenin to target genomic loci is the hallmark of canonical Wnt signaling, β-catenin and the family of TCF DNA-binding proteins are part of a regulatory system used for context-dependent control of distinct genetic programs and stage- or tissue-specific transcriptional responses [11, 39]. Therefore, we proceeded to examine the global transcriptional changes resulting from the Wnt-3a treatment of monocytes.

### Wnt-3a modifies the levels of monocyte-secreted immune proteins

**Fig. 4:**
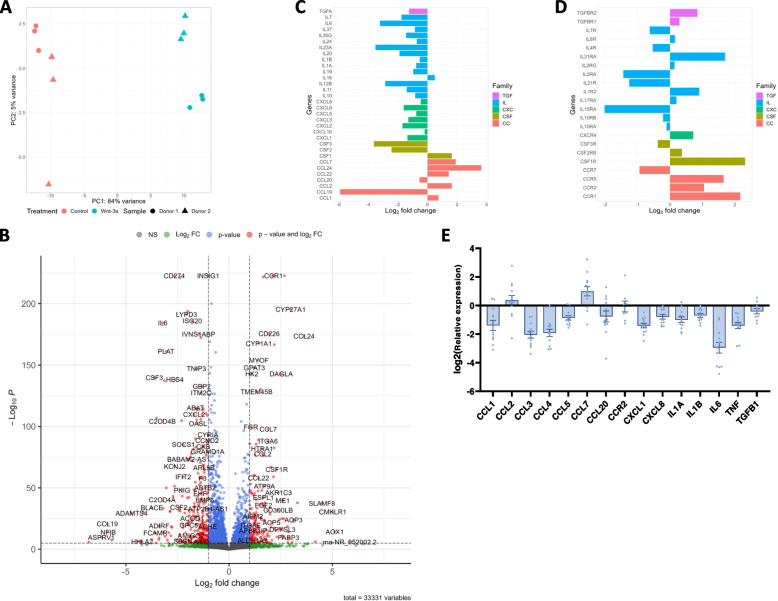
Wnt-3a modifies the levels of monocyte-secreted immune proteins. Freshly isolated monocytes (from two donors) were cultured (in triplicates) for 8 h in the presence of control or Wnt-3a conditioned media (CM) for RNA sequencing. **A** Principal component analysis based on entire gene expression patterns, with batch effect removal to standardize the different donors. **B** Volcano plot of all genes based on differential expression analysis between control- and Wnt-treated monocytes. Significantly regulated genes are in red on either side (as defined by adjusted *p*-value < 0.05 and absolute fold change > 2). **C, D** Summary of Log2 Fold Change of significantly regulated cytokine and chemokine ligands **C** and receptors **D**. **E** Similarly treated monocytes from multiple blood samples were lysed for RT-qPCR assays of the indicated cytokines and chemokines

RNA sequencing (RNA-Seq) of control- and Wnt-3a-treated human primary monocytes from two donors, cultured in triplicates, revealed distinct gene expression profiles in treated versus untreated cells (Fig. 4A). These were compared by using Illumina Human arrays (composed of 33,331 probe sets to measure human mRNA; Supplementary Table 1). The results identified 914 differentially expressed genes, with 362 significantly upregulated and 552 significantly downregulated after treatment (as defined by adjusted *p*-value < 0.05 and absolute fold change > 2; Supplementary Table 1). The differential expression analysis is presented as a volcano plot (Fig. 4B). Gene ontology examination revealed significant changes in pathways related to chemokine and cytokine production and migration pathways (Supplementary Table 1). Interestingly, most of the genes of chemokine and cytokine protein families, with the exception of some CCL family genes, were downregulated (Fig. 4C). In contrast, although there were significant changes in the expression of the chemokine and cytokine receptors, no apparent inclination was observed (Fig. 4D). In order to confirm the RNA-seq results, we performed numerous RT-qPCR experiments on Wnt-3a-treated and -untreated monocytes from blood samples donated by multiple healthy individuals (Fig. 4E). The results reinforce the RNA-seq data as they also reveal a clear tendency of chemokines and cytokines downregulation following treatment (12 out of 15 genes tested). This finding and the gene ontology analysis, which implicated genes involved in cell migration, specifically in monocyte migration, led us to explore the possibility that Wnt signaling may regulate chemokine secretion from circulating monocytes.

### Wnt-3a affects monocyte chemokine secretion

**Fig. 5:**
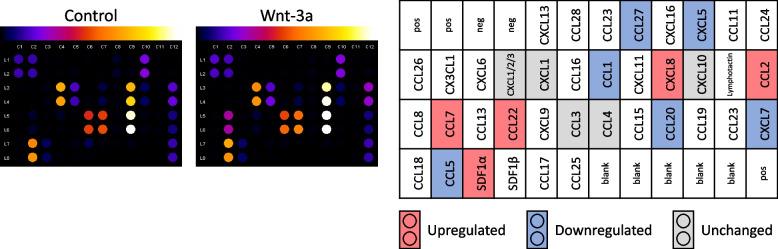
Wnt-3a affects chemokine secretion from monocytes. Media collected from the treated monocytes from two donors were centrifuged twice to remove the cells, and subjected to a chemokine membrane array (see methods section). Analysis of a representative membrane is shown in the two left panels. A summary of the changes in the chemokines detected in the two experiments is presented on the right: upregulated (red), downregulated (blue), and unchanged (grey) proteins. Original images and analyses are presented in Fig. S4

The effect of Wnt signaling on the pattern of monocyte secretion was examined by culturing isolated monocytes with or without Wnt-3a and analyzing the collected cell media with a commercial chemokine array (Fig. 5, Fig. S4). Out of the 38 ligands tested, 16 chemokines were clearly detected in monocyte media, of which 5 were upregulated (Fig. 5; red), 6 downregulated (Fig. 5; blue), and an additional 5 unaffected by Wnt-3a treatment (Fig. 5; gray). The results mostly support the RNA data (shown in Fig. 4E). In addition, as expected from the RNA-seq analysis, a noticeable number of the genes and proteins affected by Wnt activation proved to be associated with monocyte chemoattraction. These include CCL2 and CCL7 (upregulated), CCL5 (downregulated), and CCL3 and CCL4 (decreased expression of RNA but stable secretion of protein). In view of the increase in the secretion of CCL7 and the potent CCL2, along with the decrease in the secretion of other weaker monocyte chemoattractants, we examined the cumulative effect of these changes on monocyte migration.

### Treating monocytes with Wnt-3a induces cell migration and CCL2 production

**Fig. 6:**
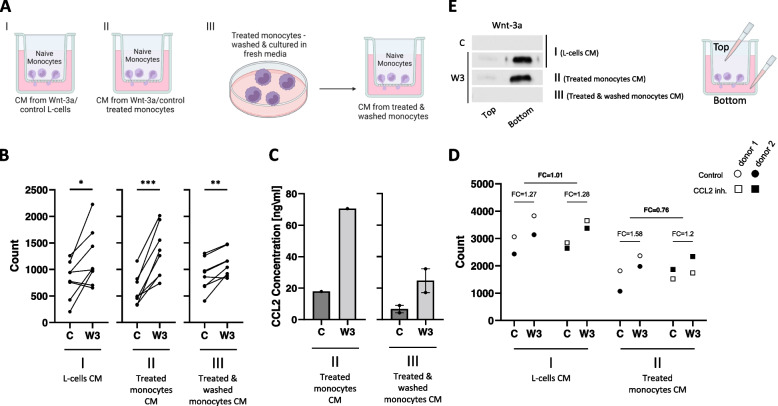
Treating monocytes with Wnt-3a induces cell migration and CCL2 production. Naïve monocytes were plated in the top chamber of a transwell and exposed to different conditioned media (CM). Cells that crossed the transwell were collected from the bottom well and counted by flow cytometry. **A** Schematic illustration of experimental design. Naïve monocytes were exposed to Wnt-3a/control CM obtained from L-cells for 2 h (I). Naïve monocytes were exposed to Wnt-3a/control CM obtained from monocytes treated with Wnt-3a/control for 8 h (II). Treated monocytes (as in II) were washed and transferred to fresh RPMI medium for an additional 1 h. CM was collected and used to treat naïve monocytes in the transwell assay (III). **B** Flow cytometry counts of cells crossing the transwell. *P-value = 0.0307, ***P-value = 0.0003 and **P-value = 0.0045 for paired t-tests. Y-axis represents the absolute cell count. **C** ELISA assays of CCL2 concentration in media used for the indicated experiments. Protein concentrations were calculated by regression from a standard curve. **D** Flow cytometry counts of cells crossing the transwell. Y-axis represents the absolute cell count. X-axis represents the media used as in I and II of the experimental design, with or without 2 μg/ml CCL2 inhibitor, marked as circles and squares, respectively. The white and black shapes represent each donor. FC – average fold change of the marked pairs. **E** A representative blot of media collected from the top and bottom chambers of the different experiments (as indicated), at the end of the transwell incubation period. Media were centrifuged to remove cells and analyzed by western blot using a specific anti-Wnt-3a antibody. C – control/control-treated media, W3 – Wnt-3a/Wnt-3a-treated media

The finding that Wnt signaling leads to changes in monocyte self-chemoattractant secretion prompted us to examine the effect of Wnt-3a on monocyte migration, specifically with respect to Wnt-induced secretion of chemokines. First, we established the ability of Wnt-3a itself to induce monocyte migration using transwell migration assays as illustrated (Fig. 6A.I). Freshly isolated monocytes were plated onto transwell inserts, which were then immersed in control or Wnt-3a conditioned media (CM, see Material and Methods section). The results revealed a moderate increase in monocyte migration along a Wnt-3a gradient (Fig. 6B.I). Western blot analysis of cell-free CM taken from the top or bottom of the transwell (see icon in Fig. 6E) revealed the presence of Wnt-3a only in the Wnt-3a CM, with minimal diffusion from bottom to top (Fig. 6E.I). Next, we exposed freshly isolated naïve monocytes to monocyte-derived cell-free CM obtained from Wnt-3a- or control-treated monocytes (henceforth: W3-Ms and C-Ms) collected after 8 h (illustration – Fig. 6A.II). This CM contained the additional chemokines that were secreted by monocytes in response to the different treatments, including elevated levels of CCL2 in CM derived from W3-Ms (Fig. 6C.II). The presence of the Wnt-3a ligand was also confirmed (Fig. 6E.II). Figure 6B.II demonstrates that, under these conditions, monocyte migration was significantly increased in response to W3-Ms-derived CM, possibly due to the rise in CCL2 secreted by the treated monocytes. However, this experiment (exp. II) could not differentiate between the effect of the Wnt-3a present in the CM and any chemoattractants secreted by the W3-Ms. In order to resolve this issue, W3-Ms and C-Ms were transferred to fresh culture media for an additional 1 h before collection of CM (illustration – Fig. 6A.III). This experiment relies on the data depicted above (Fig. 4) where the changes in the mRNA levels of the relevant chemokines led us to expect that the Wnt-treated monocytes would continue to secrete the same components even after removal of the Wnt stimulus. Our results indicate that these CM, which contain no Wnt-3a (Fig. 6E.III), increases the migration of naïve monocytes (Fig. 6B.III), supporting the observation of upregulated CCL2 (Fig. 6C.III). This last finding (exp. III) suggests that Wnt activation alters monocyte secretion patterns causing them to upsurge their auto-chemoattraction properties, in a way that increases the migration of untreated monocytes. Similar experiments were conducted with freshly isolated PBMCs (Fig. S5), where the migration patterns of monocytes and lymphocytes were compared (Fig. S5B, C). The results indicated that the Wnt-3a effect is unique to the monocytic subgroup, thereby further demonstrating the specific nature of Wnt signaling in distinct cell populations.

To further assess the Wnt-3a effect on migration, we repeated the experiment in Fig. 6B.I-II, with and without neutralizing CCL2 antibodies. The results show that, as expected, in the absence of CCL2 (exp. I), the moderate effect of Wnt-3a on cell migration was unchanged by CCL2 inhibition (Fig. 6D. pair I, fold change = 1.01). The migration pattern of monocytes exposed to CM containing both Wnt-3a and CCL2 (exp. II) was accelerated in the control but repressed following the addition of CCL2 inhibitors (Fig. 6D. pair II, fold change = 0.76). This finding further supports the notion that the enhanced migration due to Wnt-induced monocyte secretion was at least partially mediated by CCL2.

### The inflammatory state of patients with rheumatic joint diseases affects monocyte reaction to Wnt-3a stimulus

**Fig. 7:**
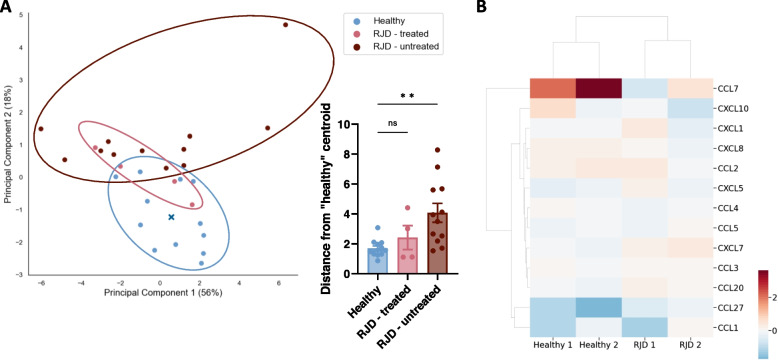
The inflammatory state of patients with rheumatic joint diseases affects monocyte reaction to Wnt-3a stimulus. **A** Principal component analyses (PCA) based on RT-qPCR data (presented in Fig. S6B) of Wnt-3a-treated monocytes (treated as in Fig. 4E). The blue X represents the centroid of the healthy donor samples, calculated as the average of the x and y coordinates of all the blue dots on the PCA graph. The bar graphs represent a calculation of distance of each dot from the healthy donor centroid, based on x,y coordinates. One-way ANOVA with Dunnett's multiple comparisons test to healthy control were employed. Healthy donors (blue), treated RJD patients (pink) and RJD patients naïve to treatment (maroon). **P-value = 0.0024, ns = 0.6717. **B** Media were collected from the treated monocytes of two RJD patients naïve to immunosuppressive treatment. The media were subjected to a chemokine membrane array and analyzed, as performed with media of healthy donors in Fig. 5. The heatmap was created using numerical data of both the data of healthy donors represented in Fig. 5 (Healthy 1&2) and the data of the two RJD patients (RJD 1&2). The fold change (FC) of signal ratio of Wnt-treated to control-treated monocyte is shown as a heatmap (log2(FC)), where upregulated genes are represented in red, downregulated in blue, and unchanged in white. Hierarchical clustering was performed on both the donors on the X-axis and the secreted proteins on the Y-axis. Only proteins clearly detected across all four membranes are represented. Original images and analyses of all 4 donors are presented in Fig. S4, Fig. S7, and the raw figure data (Supplementary file 3). RJD – rheumatic joint diseases

Wnt-3a is known to stimulate immunological processes. Therefore, we evaluated the transcription of immune effectors in monocytes of patients affected with different inflammatory states in response to Wnt-3a treatment (Fig. S6A, Supplementary Table 2). In order to assess the transcription pattern, principal component analysis (PCA) was performed based on expression of 13 genes of chosen cytokines and chemokines (Fig. S6C). The resulting scatter plot revealed a cluster of healthy controls, whereas the patient data exhibit a dispersed pattern. Interestingly, patients with psoriatic arthritis and rheumatoid arthritis (hence referred to as rheumatic joint diseases; RJD), demonstrated a scatter pattern that was significantly distanced from the healthy patients, as compared to patients without RJD (Fig. S6C; graph). Further analysis of these RJD patient data revealed a significant difference between patients treated with immunosuppressive agents and untreated patients (Fig. S6B, Fig. 7A, Fig. S6D for annotations, and Supplementary Table 2).

The differences in the monocytic transcription between healthy donors and RJD patients following Wnt-3a stimulus led us to investigate chemokine secretion. We repeated the experiment in Fig. 5 using media collected from Wnt- and control-treated monocytes of two RJD patients naïve to immunosuppressive treatment (Fig. 7B, Fig. S7). As depicted in the heat map (Fig. 7B), chemokines that were clearly visualized in all donors were assessed for up- and downregulation, revealing that some protein expression patterns were unaltered in RJD patients, whereas other proteins showed variations between monocytes of RJD patients and healthy donors. The strong upregulation of CCL7 in healthy donors was reversed in the RJD monocytes, a pattern similar to the changes in CXCL8 secretion. On the other hand, the previously downregulated CXCL7 and CCL20 are upregulated in RJD patients.

## Discussion

Monocytes are a core component of immune reactions, and though many studies focus on monocyte-derived differentiated cells [17, 40], monocytes can also function with minimal differentiation [41], and the identification of molecules that regulate their activity is of great importance. Revealing the root mechanisms inducing immune reactions triggering diseases is a long-standing challenge in medicine. Thus, understanding monocyte responses to multi-faceted pathways has the potential to accelerate treatment design and improve patient care.

In this study, we demonstrate that Wnt-3a, an endogenous glycoprotein that activates the canonical Wnt signaling pathway, induces changes in monocyte secretion and migration patterns without affecting the classical Wnt target genes. This result is in accordance with additional studies demonstrating that Wnt-3a does not lead to TCF-promoter activity or the expression of classical canonical Wnt signaling target genes in T lymphocytes or THP-1 monocyte cells [35, 36, 42]. It also supports the notion that the expression of Wnt target genes is highly context-dependent, and the combination of transcription factors produces a particular outcome that depends on the specific cellular and temporal constellations [43]. Indeed, although shown to induce the upregulation of β-catenin in vascular smooth muscle cells, Wnt-3a is involved in the activation of noncanonical ROCK/JNK signaling rather than affecting classical canonical Wnt targets [44].

Although Wnt-3a was reported to inhibit monocyte migration [36], our results suggest that Wnt-3a facilitates monocyte chemoattraction. This discrepancy can be explained by different experimental conditions. Here, we employed methodologies ensuring that the monocytes remained undifferentiated by using cell-repellent plates and shorter culture times. Importantly, our data reveal that Wnt-3a stimulates monocytes to produce the potent self-chemoattractant CCL2. Although direct detection of CCL2 protein was performed only on two samples (Fig. 6C), this finding is supported by our qPCR results (Fig. 4E), the protein secretion array (Fig. 5) and the inhibition through CCL2 neutralization (Fig. 6D). A similar response was observed in neutrophils isolated from peripheral blood that react to Wnt-5a by increased migration as well as CCL2 production [45]. Interestingly, CCL2 was reported to be a target gene of the canonical Wnt pathway in breast cancer [46], and the interaction between β-Catenin and CCL2 is thought to promote monocyte migration toward glioblastoma cells [47]. Indeed, the effect of Wnt-3a on monocyte migration may be mediated via Wnt-stimulated secretion of CCL2, as it is inhibited by the neutralization of CCL2 in the media. The increase in the migration rate of the Wnt-3a-treated monocytes can also be attributed to other cellular modifications. For example, additional genes that may trigger cell migration were upregulated by Wnt-3a stimulus according to our RNA-seq data, such as CMKLR and AQP3 [48]. Alternatively, it is possible that some monocyte chemoattractants were secreted into the CM within the 8-h incubation time as was previously demonstrated. IL-1β, IL-6 and IL-8 were detected in monocytes after only 4 h following various stimuli [49]. Collectively, the data suggest that the Wnt pathway may regulate the immune response by inducing changes in the secretory repertoire and leukocyte migration, possibly through crosstalk with the NF-κB pathway, which controls the production of growth factors, chemokines including CCL2, and cytokines in immune cells [50]. Interestingly, a recent study found that Wnt/RYK signaling through β-catenin and NF-κB is part of a safeguard mechanism against mesenchymal cell death, excessive inflammatory cytokine production, and inflammatory cell recruitment and accumulation [51]. Indeed, our analysis shows that the RYK receptor is expressed in monocytes. A functional correlation between aberrant Wnt signaling, monocytes, and inflammation has been demonstrated in certain tissues [52], where dysfunctional monocytes were reported to exhibit abnormal Wnt transcription compared to healthy subjects [53]. Monocyte involvement in inflammation is also manifested in atherosclerosis, where monocytes adhere to endothelial cells, a process that was recently connected to activation of Wnt signaling [54].

Our results show that monocytes of patients affected with psoriatic arthritis and rheumatoid arthritis (referred to in this paper as RJD) react differently to Wnt-3a stimulus in terms of chemokine and cytokine expression. However, following the accepted RJD treatment, patients' monocytes exhibit a healthier expression of these immune response effectors. Monocyte migration in response to CCL2 is an integral part of the pathophysiology seen in rheumatic diseases [31, 55], pathologies that are known to also involve Wnt signaling [56]. Studies showed that various chemokines participated in RJD pathogenesis: rheumatoid arthritis patients exhibit increased levels of plasma CCL2, CCL3, CCL4, and CXCL10 [57] and psoriatic arthritis patients also exhibited high values of CCL2 and CXCL10 [58]. Thus, as our findings demonstrate a Wnt-mediated effect on migration and CCL2 levels, studying the function of bloodstream Wnt signaling in both homeostasis and abnormal conditions may shed light on pathologies involving circulating WBCs. Our findings also demonstrate downregulation of monocyte-secreted pro-inflammatory cytokines in response to Wnt-3a, it will be interesting to assess the cell reaction to Wnt in these diseases, where treatments targeting such cytokines have already proven effective [59, 60]. As a first step, we compared the chemokine secretion patterns, in response to Wnt-3a stimulation, between monocytes of healthy donors and RJD patients. Patient cells treated with Wnt-3a demonstrate enhanced secretion of CCL20, a known chemokine of lymphocytes found to be correlated with RJD [61]. Conversely, while healthy donor cells responded with an increase in the secretion of CCL7 and CCL2, both strong monocyte chemoattractants, RJD patient cells did not. These results suggest that Wnt signaling has a differential effect on healthy monocytes as compared to RJD patient monocytes, potentially shifting the response from an innate inflammation mediated by monocytes through CCL2 and CCL7 towards an adaptive immune response mediated by CCL20.

Healthy donors were selected from students and staff volunteers, which resulted in a lower mean age compared to the RJD patients. In addition, due to technical issues, patient samples were sometimes stored overnight before carrying out the experiments. These limitations could have affected our analyses.

## Conclusion

Our study reveals an intricate relationship between Wnt signaling and circulating human monocytes. We demonstrated that Wnt-3a can act as a stimulator of monocyte-driven immune processes, inducing the secretion of cytokines and chemokines that enhance monocyte migration. Furthermore, our findings suggest that dysregulation of the Wnt signaling pathway may contribute to the pathogenesis of rheumatic joint diseases, highlighting the importance of further research in this area. This connection may provide the basis for a novel concept of circulation signaling pathways that modulate systemic changes, thus affecting core biological processes at sites of inflammation.

## Materials & methods

### Solutions and buffers

M2 lysis buffer (for western blot analysis): 100 mM NaCl, 50 mM Tris pH 7.5, 1% Triton X-100, 2 mM ethylenediamine tetraacetic acid (EDTA).

SDS sample buffer × 5: 3% 3 M Tris–HCl pH 6.8, 25% Glycerol, 10% SDS 20%, 5% β-mercaptoethanol, 10% of 1% Bromophenol Blue in water.

Blocking buffer (for immunofluorescence): 5% fetal calf serum (FCS) in phosphate buffered saline (PBS).

Blocking buffer (for CCL2 ELISA): 2% bovine serum albumin (BSA) in PBS.

Wash buffer (for CCL2 ELISA): 5% 1M Tris-base, 0.2% Tween, 0.2% HCl in water.

### Cell culture and conditioned media preparation

L-Wnt-3a cells, L cells, and THP-1 cells were propagated according to the ATCC guidelines. Conditioned media (CM) from L-cells (L, L-Wnt-3a) were produced according to the ATCC protocols. Low passage THP-1 cells were induced to differentiate into macrophages by a 24-h incubation with 160 ng/ml phorbol 12-myristate 13-acetate (PMA). Isolated primary human monocytes were cultured at 1–3*10^6^ cell/ml with 80% CM and 20% RPMI-1640 (both containing 10% FBS and 1% Penicillin/Streptomycin) at 37°C in 5% CO_2_ in cell repellent 6-well plates (Greiner Bio-One 657970) for the indicated periods.

### Whole blood separation

PBMC separation was performed as previously described [22] with an additional washing step that included centrifugation of the cells at 300 g for 10 min, and washing in PBS at 200 g to better eliminate platelets.

Plasma separation was performed as previously described [22].

Monocytes were negatively selected on magnetic columns according to the manufacturer’s protocol (Miltenyi Biotec, Classical Monocyte Isolation Kit 130–117-337, LS Columns 130–042-401).

### Real-time quantitative PCR

RNA extraction (Direct-zol RNA MiniPrep kit) and cDNA synthesis (iScript cDNA Synthesis Kit, Bio-Rad) were done according to the manufacturer’s protocols. RT-qPCR was performed as described [22] using the ΔΔCq technique: β-actin was used as a housekeeping gene and Wnt-treated samples were normalized to the control.

Primers were based on previous publications or designed based on the UCSC genome browser [62] sequence using the ApE program [63] and verified using the USCS In-Silico PCR Tool [56]. The primers for the amplification of the specific cDNA sequences were:

**Table:** 

Gene	Primer	Sequence (5’- > 3’)	Amplicon size (bp)
AXIN2	Forward	CGAGCTCAGCAAAAAGGGAAAT	114
	Reverse	TACATCGGGAGCACCGTCTCAT	
CCND1	Forward	CTGTGCATCTACACCGACAA	78
	Reverse	CTTGAGCTTGTTCACCAGGA	
LEF1	Forward	AGACAAGCACAAACCTCTCAG	130
	Reverse	TCATTATGTACCCGGAATAACTC	
MYC	Forward	CTGGTGCTCCATGAGGAGAC	142
	Reverse	CAGCAGAAGGTGATCCAGACTC	
SOX9	Forward	ACTTGCACAACGCCGAG	140
	Reverse	CTGGTACTTGTAATCCGGGTG	
CCL1	Forward	CTGAGGGCAATCCTGTGTTAC	116
	Reverse	TGAACCCATCCAACTGTGTC	
CCL2	Forward	AGCAGCCACCTTCATTCC	159
	Reverse	AAGATCACAGCTTCTTTGGGAC	
CCL3	Forward	CTGCTTCAGCTACACCTCC	167
	Reverse	CCAGGTCGCTGACATATTTC	
CCL4	Forward	TCTCAGCACCAATGGGCTC	160
	Reverse	GCACAGACTTGCTTGCTTC	
CCL5	Forward	GCTGTCATCCTCATTGCTACTG	152
	Reverse	TTGGAGCACTTGCCACTG	
CCL7	Forward	ACAGAAGGACCACCAGTAGCCA	117
	Reverse	GGTGCTTCATAAAGTCCTGGACC	
CCL20	Forward	TGATGTCAGTGCTGCTACTCC	143
	Reverse	GATGTCACAGCCTTCATTGG	
CCR2	Forward	CTGTGAAAGCACCAGTCAAC	196
	Reverse	TTCTTTCCTGGTCTCACTCC	
CXCL1	Forward	AGGGAATTCACCCCAAGAAC	204
	Reverse	CACCAGTGAGCTTCCTCCTC	
CXCL8	Forward	TCTTGGCAGCCTTCCTGATT	171
	Reverse	TTTCTGTGTTGGCGCAGTGT	
IL1A	Forward	GGTTGAGTTTAAGCCAATCCA	89
	Reverse	TGCTGACCTAGGCTTGATGA	
IL1B	Forward	AGCTGATGGCCCTAAACAGA	93
	Reverse	TGGTGGTCGGAGATTCGTAG	
IL6	Forward	ACTCACCTCTTCAGAACGAATTG	149
	Reverse	CCATCTTTGGAAGGTTCAGGTTG	
TNF	Forward	CAGCCTCTTCTCCTTCCTGA	123
	Reverse	GCCAGAGGGCTGATTAGAGA	
TGFB1	Forward	AGCCCTGGACACCAACTATTGC	136
	Reverse	GAGGCAGAAGTTGGCATGGTAG	
ACTB	Forward	CCTGGCACCCAGCACAAT	144
	Reverse	GGGCCGGACTCGTCATACT	

### Library construction and RNA sequencing

Total RNA was extracted us ing the Direct-zol RNA MiniPrep kit. Replicates of high RNA integrity (RIN ≥ 7.8) were processed. RNA-seq libraries were prepared and sequenced at the Genomics Research Unit, at the Life Sciences Inter-Departmental Research Facility Unit, Tel-Aviv University, Israel. Libraries were prepared using the NEBNext Ultra II RNA Library Prep kit with the NEBNext Poly(A) mRNA Magnetic Isolation Module, starting with ~ 500 ng of total RNA. Ten PCR cycles were performed during library amplification. Libraries were quantified by Qubit (Thermo Fisher Scientific) and TapeStation (Agilent), and sequenced on a NextSeq 500 instrument using a NextSeq 500/550 High Output Kit v2.5 (75 Cycles) kit.

### RNA sequencing initial analysis and quality control

Twelve FastQ files were uploaded to Partek Flow (Build version 10.0.21.1116; https://www.partek.com) for processing. Poly-A/T stretches and Illumina adapters were trimmed from the reads. Resulting reads with Phred score < 20 were filtered. Reads were mapped to the human GRCh38.p13 reference genome using STAR 2.7.8a with default parameters [64] to reveal ~ 20 million reads per sample. Quantification to annotation model was performed using Partek Expectation/Maximization (E/M) algorithm [65] obtaining 33,331 genes. Initial analysis and quality control were performed at the Bioinformatics Unit, The Life Sciences Inter-Departmental Research Facility Unit, Tel-Aviv University, Israel.

### RNA sequencing data analysis

The count matrix was analyzed using R language for differentially expressed (DE) genes using *DESeq2* [66]. Principal component analysis (PCA) was performed using *DESeq2* with removal of batch effects using *limma* [67]. The lists of DE genes were analyzed using *goseq* for gene ontology analysis [68]. A volcano plot of DE was plotted by *EnhancedVolcano* [69]. Other plots were created using *ggplot2* [70].

### Analysis of single-cell RNA sequencing data obtained from independent sources

Single-cell RNA sequencing data obtained from 10X genomics, and analyzed using R language with *Seurat* using the “pbmc3k” tutorial [71]. Data and UMAP analyses available at the Human Protein Atlas website (proteinatlas.org) were used directly [72] – these data were not available for independent analysis.

10X genomics data: 3k PBMCs from a Healthy Donor, Cell Ranger 1.1.0, 10 × Genomics, 2016, May 26. https://www.10xgenomics.com/resources/datasets/3-k-pbm-cs-from-a-healthy-donor-1-standard-1-1-0

### Enzyme-linked immunoassay (ELISA) for Wnt-3a and CCL2

Wnt-3a protein levels of platelet-poor plasma of healthy donors were analyzed using Human WNT3A ELISA Kit (LSBio LS-F12973) following the manufacturer’s protocol. Plasma was diluted 1:1 or 1:4 in PBS for optimal fit to the standard curve.

CCL2 protein levels in monocyte culture media were measured using mouse anti-human CCL2 (PeproTech 500-M71) for coating and biotinylated rabbit anti-human CCL2 (PeproTech 500-P34Bt) for detection. The samples were diluted 1:1 and 1:9 in blocking buffer. Following incubation with streptavidin–horseradish peroxidase, TMB/E was added (Chemicon, Temecula, CA, USA). The reaction was stopped by addition of 0.18 M sulfuric acid. Recombinant human CCL2 (PeproTech 300–04) was used to create a standard curve.

Optical densities were measured at 450 nm by Epoch Microplate Spectrophotometer (BioTek). Data analysis was performed using GainData based on a 4-Parameter Logistic Regression (Arigo Biolaboratories Corporation. arigo’s ELISA Calculator https://www.arigobio.com/ELISA-calculator.).

### Western blot analysis

Protein extraction, SDS-PAGE separation, and visualization were performed as described [73], except for the detection step (Immobilon Forte, Millipore WBLUF0500) and band intensity quantification (ImageJ software). Western blot analysis of media was performed similarly, without a lysis step (media were directly diluted with sample buffer X5 and denaturized by heating).

Antibodies: mouse anti-Actin (MP biomedical 69100, 1:10,000), mouse anti-β-catenin (BD 610154, 1:2000), rabbit anti-c-Myc (Santa Cruz 788, 1:500), rabbit anti-Frizzled (Santa Cruz Biotechnology 9169, 1:500), mouse anti-GSK3 (Santa Cruz Biotechnology 7291, 1:500), rabbit anti-LEF1 (Abcam 137872, 1:1000), mouse anti-LRP5 (Abcam 129357, 1:500), rabbit anti-LRP6 (Abcam 134146, 1:1000), mouse anti-TCF4 (Millipore 05–511, 1:1000), rabbit anti-Wnt-3a (Cell Signaling 2721, 1:3000), Goat anti-mouse HRP (Jackson ImmunoResearch 115–035-003, 1:10000), Goat anti-rabbit HRP (Jackson ImmunoResearch 111–035-144, 1:10000).

### Immunofluorescence

Cells were fixed in PBS containing 4% paraformaldehyde for 20 min, permeabilized with blocking buffer containing 0.01% Triton X-100 for 10 min, and blocked for 1 h in blocking buffer. Cells were incubated with primary and secondary antibodies diluted in blocking buffer containing FcR blocking reagent (Miltenyi Biotec 130–059-901) for 1.5 h and 45 min, respectively. 4′,6-diamidino-2-phenylindole (DAPI) was used to stain cell nuclei. After each step, the samples were washed with PBS, and the tubes were centrifuged at 600 g for 4 min at 4°C. All incubations were performed at room temperature while shaking the tubes.

Cells were resuspended in fluorescence mounting reagent (GBI Labs E18-18) and fixed to a slide with a cover slip. Slides were visualized by confocal microscopy, with at least 10 fields taken for each slide.

Images were quantified by a custom Python script (Supplementary file 4). Briefly, after identification and elimination of the background signal, the script calculated the mean intensity of each field and standardized it to the average DAPI value of the entire slide (to eliminate random intensity changes between images). The nuclear localization script identifies the location of non-background DAPI pixels, and calculates the β-catenin and DAPI intensities of those pixels, followed by standardizing the β-catenin signal to the nuclear intensity.

Antibodies: rabbit anti-Axin2 (Cell Signaling 76G6, 1:150) mouse anti-β-catenin (BD 610154, 1:150), rabbit anti-c-Myc (Santa Cruz 788, 1: 150), mouse anti-Cyclin D (Santa Cruz 20044, 1:50), rabbit anti-LEF1 (Abcam 137872, 1:150), rabbit anti-Sox9 (Millipore AB5535, 1:150), Anti-mouse IgG rhodamine red (Jackson ImmunoResearch 715–295-150, 1:500), anti-rabbit IgG Alexa Fluor 488 (Invitrogen A11034, 1:500).

### Chemokine membrane array

Monocytes from two healthy donors and two patients were cultured as described for 8 h. The collected media samples were centrifuged twice at 600 g for 4 min at 4°C to achieve cell-free media. Chemokines in the media were detected by a chemokine antibody array (RayBio AAH-CHE-1–4). Images were obtained using Fusion Pulse (Vilber) using the serial option to obtain images every 2 s. Raw numerical densitometry was obtained for multiple images of each experiment using Protein Array Analyzer for ImageJ (Carpentier G. available online: http://rsb.info.nih.gov/ij/macros/toolsets/ProteinArrayAnalyzer.txt). Data from the best signal image of each protein were used: before signal saturation on either the treatment or the control array, and before saturation of any positive control. Background signal substruction and standardization were performed in Microsoft Excel, yielding a fold change ratio of normalized Wnt signal to raw control signal, as per the manufacturer’s instructions.

### Patient sample handling and data analysis

Patient blood samples were collected into EDTA tubes and used immediately or stored as whole blood at 4°C overnight. Monocytes were isolated as described for healthy donor samples. Subsequent RNA extraction, RT-qPCR as well as media preparation and chemokine membrane analysis were also performed as described.

Data were analyzed using Python language. Data were processed using *Pandas* [74]. Plots were generated using *Matplotlib.pyplot* [75] and *Seaborn* [76]. Healthy donor and patient RT-qPCR data were used to run a Principal component analysis (PCA) using *Sklearn.preprocessing* [77]. Healthy donor and patient chemokine membrane array fold change data were used to create a heatmap using *seaborn.clustermap* without further data normalization. The coloring center was set as 0 to represent up- and downregulation of log2(fold change), and hierarchical clustering was performed on both the columns and the rows using the function’s default “average” linkage method.

### Monocyte migration assays

Cell migration was quantitated in duplicates in a 24-well Transwell using polycarbonate filters with 5 μm pores (Corning Costar 3421). Monocytes (0.3 × 10^6^ cells in 200 μl of 80% DMEM and 20% RPMI 1640, both containing 10% FBS and 1% Penicillin/Streptomycin) were added to the upper compartment of the insert. The lower well contained 600 μl of the test media (as described in Fig. 6). After a 2-h incubation at 37°C, the inserts were gently removed, the cell-containing media were gently homogenized, and 400 μl were used to count cells by flow cytometry (Cytoflex). All samples were collected during a fixed time of acquisition and gated for live singlets. The data were analyzed by Kaluza Analysis Software (Beckman Coulter).

The experiment was repeated with PBMCs in the top well (as described in Fig. S5). Monocytes could be distinguished from lymphocytes according to the physical parameters of the flow cytometry scatter (separation of the two populations based on forward scatter (FSC) and side scatter (SSC), after gating for live singlets).

In some experiments, the media were pre-incubated with the neutralizing antibody against human CCL2 (R&D Systems MAB679, 2 μg/ml) for 1.5 h at room temperature prior to being used in the lower chambers of the migration assays.

### Statistical analysis

All statistical analyses used the recommended tests and GraphPad Prism 9. No one-tailed tests were used. Data are presented as mean ± SEM.

### Supplementary Information

**Additional file 1. **RNA-seq data and analyses. Includes the count matrix, differential expression (DE) and gene ontology (GO) analyses.

**Additional file 2. **Donor information. Includes all information for healthy and patient donors.

**Additional file 3. **Raw figure data. Includes all numerical data pertaining to graph preparation, uncropped images, an annotated version of Fig. 7A and Fig. S6C, raw images and calculation template for Fig. 5 and Fig. 7.

**Additional file 4. **Python script. Includes the code for immunofluorescence quantification, as well as a step-by-step description of the program.

**Additional file 5: Fig. S1. **Canonical Wnt signaling components are expressed in monocytes (data by 10X Genomics). Analysis of single-cell RNA data by 10X Genomics, based on a Seurat tutorial (“pbmc3k”). tSNE plot of the data with annotation of cell types (upper left panel). Classical monocytes are the orange cluster labeled “CD14^+^ Mono” and non-classical monocytes are the light blue cluster labeled “CD16^+^ Mono”. Plt – platelets. NK – natural killer cells. B and T lymphocytes are abbreviated B and T, respectively. The data were further analyzed for expression of Wnt signaling components. The genes are grouped by functional categories (color-coded at the bottom). **Fig. S2.** Canonical Wnt signaling components are expressed in monocytes (analysis by Protein Atlas). UMAP analyses of Single-cell data acquired from The Human Protein Atlas. The top pair of panels represents CD14 and CD16 expression, showing cluster “c-0” to represent classical monocytes (blue circle). All other panels represent expression of Wnt-related genes. The genes are grouped by functional categories (color-coded). **Fig. S3.** Canonical Wnt components are present in THP-1 and primary monocytes. Western blot analysis of primary monocytes and THP-1 monocyte-like cells using the indicated antibodies for canonical Wnt components. **Fig. S4. **Quantification of membrane chemokine array (Healthy donors). Media collected from culture of control- and Wnt-3a-treated monocytes were centrifuged twice to remove the cells, subjected to a chemokine membrane array (top panels) and quantified (middle and bottom panels). The experiment was repeated with a second donor with similar results. A First donor – all panels. B Second donor – supplementary panels to those presented in Fig. 5. **Fig. S5. **Lymphocyte migration is not affected by Wnt-3a. A similar experiment to that in Fig. 6 using freshly isolated PBMCs in the top chambers. Cells from the bottom wells were collected, counted by flow cytometry, and identified as monocytes or lymphocytes based on the flow cytometry scatter. A A schematic illustration of the experimental setup. B, C Cell counts of monocytes B or lymphocytes C. *P-value = 0.0229, ***P-value = 0.0002, *P-value = 0.0136 for paired t-tests (from left to right). No significance was observed for the lymphocyte counts.** Fig. S6. **Data of healthy and patient monocytes treated with Wnt-3a. A, B Representation of RT-qPCR data of monocytes treated as in Fig. 4E. The continuous color legend represents Log2(Fold Change) of the gene expression of Wnt-3a-treated vs. control-treated monocytes. The tested genes are noted on the x-axis. Please note that data presented in Fig. 4E is used here as “Healthy”. A Healthy donors (blue), patients with inflammatory states other than RJD (green) and RJD patients (maroon). These data were used to produce C. B Healthy donors (blue), treated RJD patients (pink) and RJD patients naïve to treatment (maroon). These data were used to produce Fig. 7A. C Principal component analyses (PCA) based on RT-qPCR data (presented in A) of Wnt-3a-treated monocytes (treated as in Fig. 4E). The blue X represents the centroid of the healthy donor samples, calculated as the average of the x and y coordinates of all the blue dots on the PCA graph. The bar graphs represent a calculation of distance of each dot from the healthy donor centroid, based on x,y coordinates. Healthy donors (blue), patients with inflammatory states other than RJD (green) and RJD patients (maroon). *P-value = 0.0228, ns = 0.1276. D PCA plot as in Fig. 7A. Each point is annotated with the corresponding donor number. Point shape represents the donor condition: circle – healthy, triangle – rheumatoid arthritis (RA), square – psoriatic arthritis (PsA). Point color is based on immunosuppressive treatment of RJD patients: pink – treated, maroon and blue– untreated. RJD – rheumatic joint diseases. **Fig. S7. **Quantification of membrane chemokine array (RJD patients). Media collected from culture of control- and Wnt-3a-treated monocytes were centrifuged twice to remove the cells, subjected to a chemokine membrane array (top panels) and quantified (middle and bottom panels). The experiment was conducted with monocyte from two RJD patients (A, B). These data, along with the data presented in Fig. S4, were used to produce Fig. 7C. RJD – rheumatic joint diseases.

## Data Availability

The RNA-Seq datasets generated and analyzed during the current study are available in the Gene Expression Omnibus repository under accession number GSE228306 and are available at the following URL: https://www.ncbi.nlm.nih.gov/geo/query/acc.cgi?acc=GSE228306. Code for quantification of immunofluorescence signal intensity and nuclear localization is provided as a supplementary file, including a comprehensive explanation of the program steps. Code for RNA-seq analysis of our data and code for analysis of single-cell data by 10X genomics were based on commonly available R packages (as described in Materials and Methods), and will be provided upon request. All materials used in this paper are listed and are commercially available.

## Abbreviations

CCL: C–C motif chemokine ligand
CCR: C-C chemokine receptor
CM: Conditioned media
CXCL: C-X-C motif chemokine ligand
DE: Differential expression
DVL: Dishevelled
FZD: Frizzled
GSK: Glycogen synthase kinase
IL: Interleukin
LEF: Lymphoid enhancer-binding factor
LRP: Low-density lipoprotein receptor-related protein
NF-κB: Nuclear factor kappa B
PBMCs: Peripheral blood mononuclear cells
PCA: Principal component analysis
PMA: Phorbol-12-myristate-13-acetate
PsA: Psoriatic arthritis
RA: Rheumatoid arthritis
RJD: Rheumatic joint diseases
RYK: Related to receptor tyrosine kinase
TCF: Transcription factor
TGF: Transforming growth factor
TNF: Tumor necrosis factor
tSNE: t-distributed stochastic neighbor embedding
UMAP: Uniform manifold approximation and projection
WBCs: White blood cells
