# Supplementary material for: Wnt signaling regulates chemokine production and cell migration of circulating human monocytes
Source: Cell Commun Signal. 2024 Apr 16;22:229. doi: 10.1186/s12964-024-01608-8 (PMC11020454; doi:10.1186/s12964-024-01608-8)
Supplement: Supplementary file 5 — Additional file 5: Fig. S1. Canonical Wnt signaling components are expressed in monocytes (data by 10X Genomics). Analysis of single-cell RNA data by 10X Genomics, based on a Seurat tutorial (“pbmc3k”). tSNE plot of the data with annotation of cell types (upper left panel). Classical monocytes are the orange cluster labeled “CD14+ Mono” and non-classical monocytes are the light blue cluster labeled “CD16+ Mono”. Plt – platelets. NK – natural killer cells. B and T lymphocytes are abbreviated B and T, respectively. The data were further analyzed for expression of Wnt signaling components. The genes are grouped by functional categories (color-coded at the bottom). Fig. S2. Canonical Wnt signaling components are expressed in monocytes (analysis by Protein Atlas). UMAP analyses of Single-cell data acquired from The Human Protein Atlas. The top pair of panels represents CD14 and CD16 expression, showing cluster “c-0” to represent classical monocytes (blue circle). All other panels represent expression of Wnt-related genes. The genes are grouped by functional categories (color-coded). Fig. S3. Canonical Wnt components are present in THP-1 and primary monocytes. Western blot analysis of primary monocytes and THP-1 monocyte-like cells using the indicated antibodies for canonical Wnt components. Fig. S4. Quantification of membrane chemokine array (Healthy donors). Media collected from culture of control- and Wnt-3a-treated monocytes were centrifuged twice to remove the cells, subjected to a chemokine membrane array (top panels) and quantified (middle and bottom panels). The experiment was repeated with a second donor with similar results. A First donor – all panels. B Second donor – supplementary panels to those presented in Fig. 5. Fig. S5. Lymphocyte migration is not affected by Wnt-3a. A similar experiment to that in Fig. 6 using freshly isolated PBMCs in the top chambers. Cells from the bottom wells were collected, counted by flow cytometry, and identified as monocytes or [file 12964_2024_1608_MOESM5_ESM.docx]

**SUPPLEMENTARY FIGURES**

**
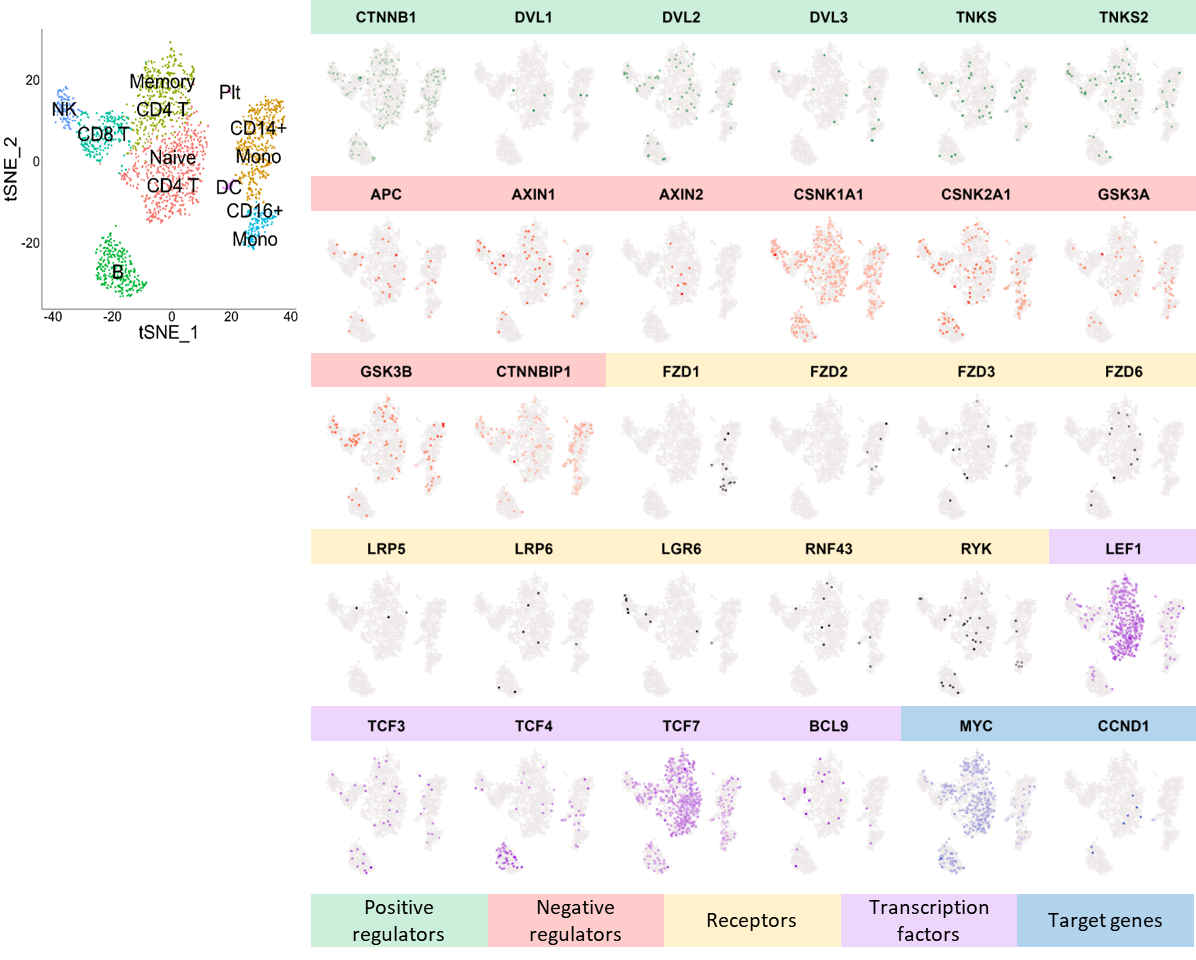
**

**Fig. S1. Canonical Wnt signaling components are expressed in monocytes (data by 10X Genomics)**. Analysis of single-cell RNA data by 10X Genomics, based on a Seurat tutorial (“pbmc3k”). tSNE plot of the data with annotation of cell types (upper left panel). Classical monocytes are the orange cluster labeled “CD14^+^ Mono” and non-classical monocytes are the light blue cluster labeled “CD16^+^ Mono”. Plt – platelets. NK – natural killer cells. B and T lymphocytes are abbreviated B and T, respectively. The data were further analyzed for expression of Wnt signaling components. The genes are grouped by functional categories (color-coded at the bottom).


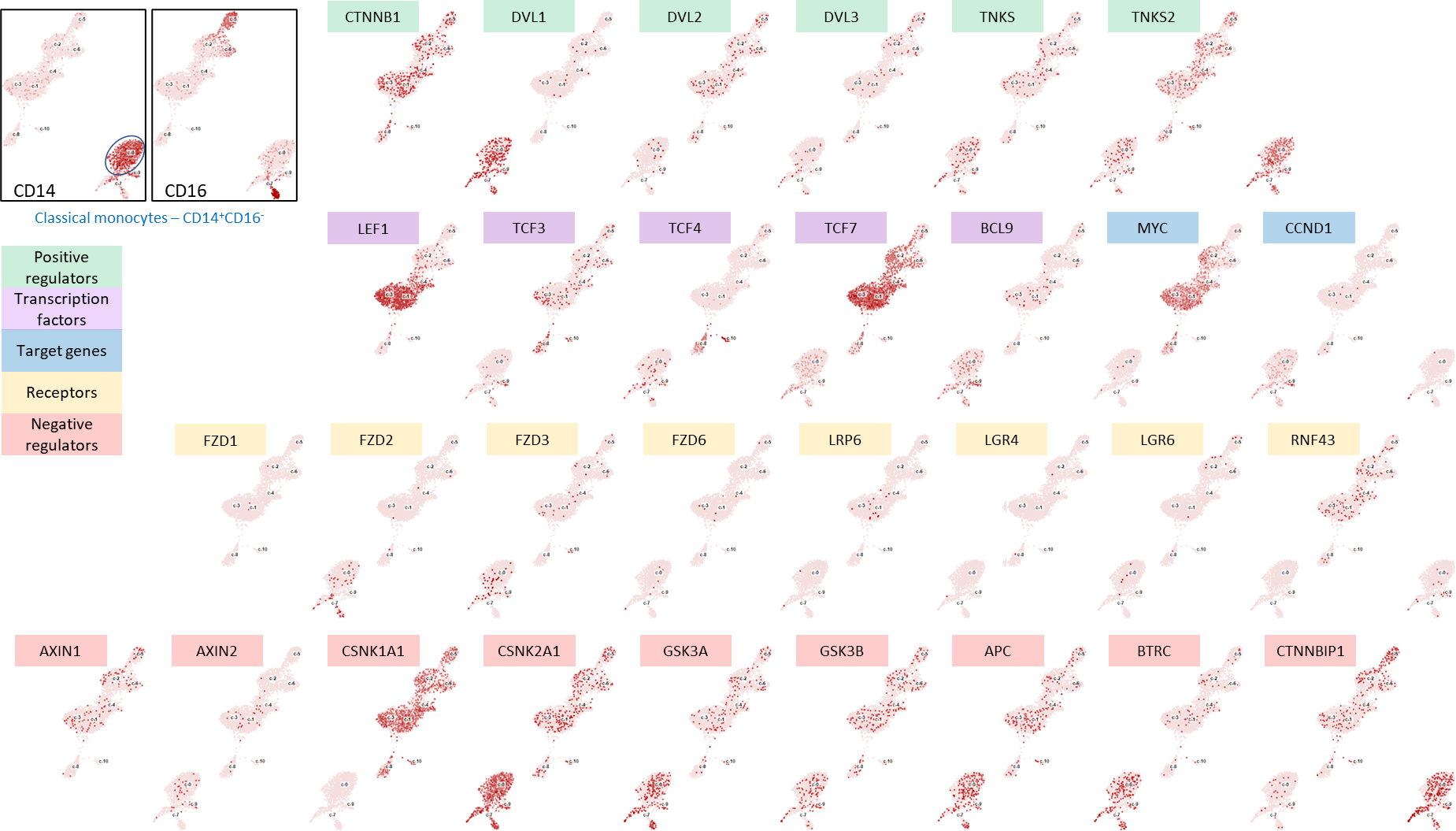


**Fig. S2.** **Canonical Wnt signaling components are expressed in monocytes (analysis by Protein Atlas)**. UMAP analyses of Single-cell data acquired from The Human Protein Atlas. The top pair of panels represents CD14 and CD16 expression, showing cluster “c-0” to represent classical monocytes (blue circle). All other panels represent expression of Wnt-related genes. The genes are grouped by functional categories (color-coded at the bottom).

**
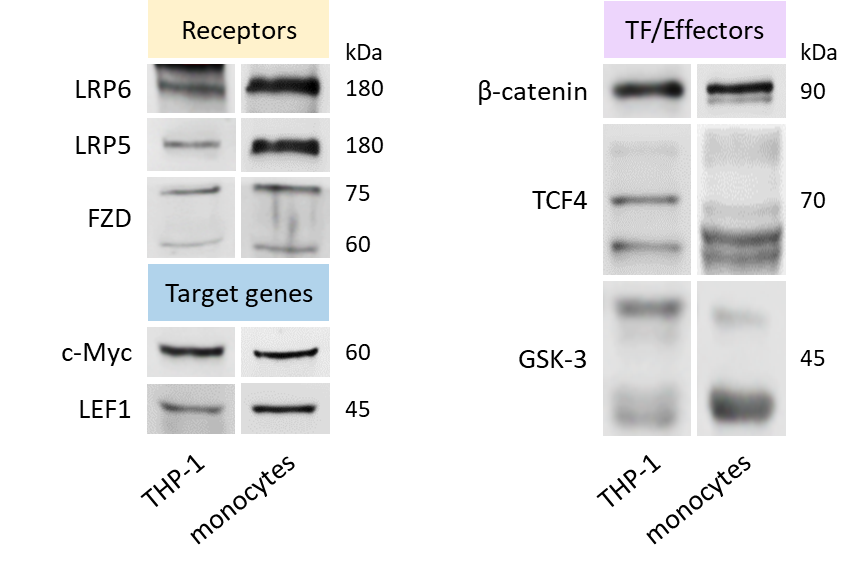
**

**Fig. S3.** **Canonical Wnt components are present in THP-1 and primary monocytes.** Western blot analysis of primary monocytes and THP-1 monocyte-like cells using the indicated antibodies for canonical Wnt components.


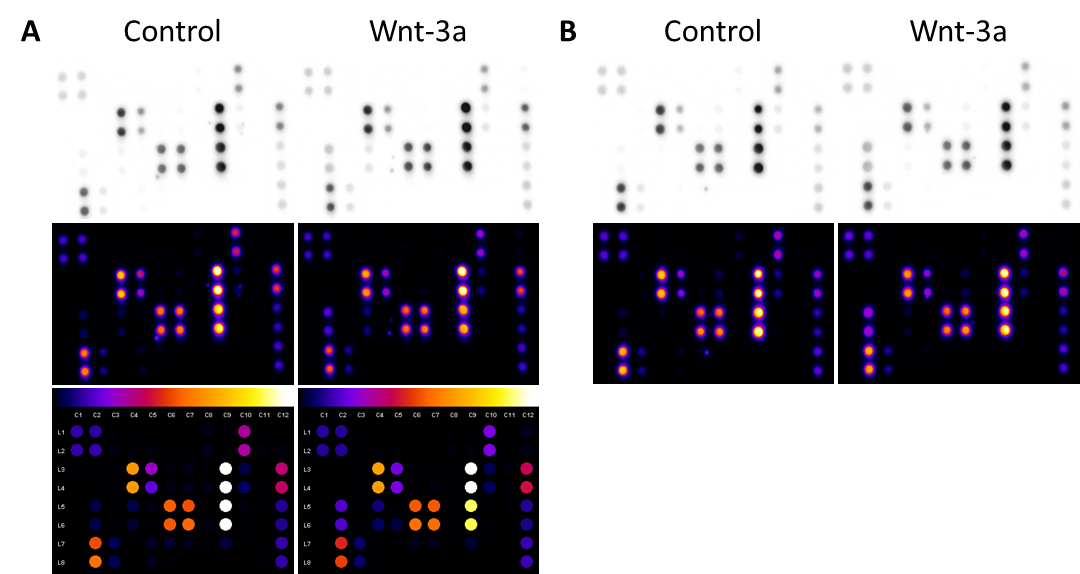


**Fig. S4. Quantification of membrane chemokine array** **(Healthy donors).** Media collected from culture of control- and Wnt-3a-treated monocytes were centrifuged twice to remove the cells, subjected to a chemokine membrane array (top panels) and quantified (middle and bottom panels). The experiment was repeated with a second donor with similar results. **A** First donor – all panels. **B** Second donor – supplementary panels to those presented in Fig. 5.


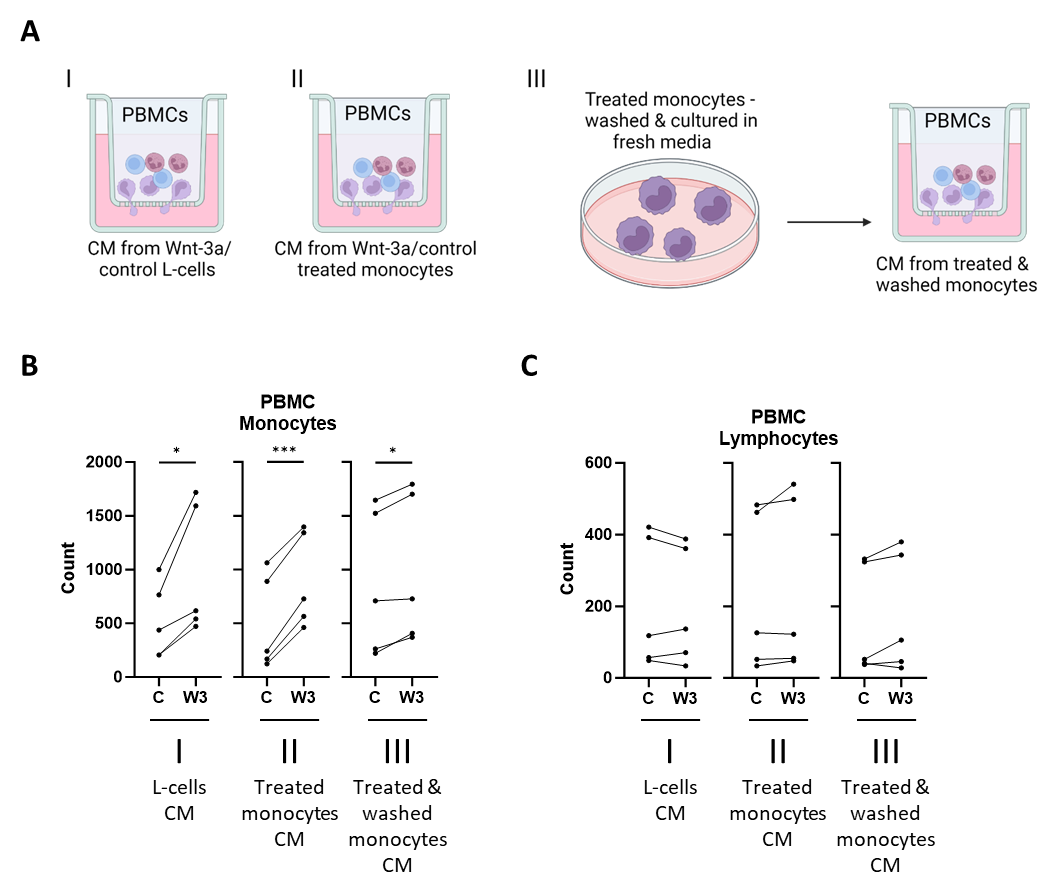


**Fig. S5. Lymphocyte migration is not affected by Wnt-3a**. A similar experiment to that in Fig. 6 using freshly isolated PBMCs in the top chambers. Cells from the bottom wells were collected, counted by flow cytometry, and identified as monocytes or lymphocytes based on the flow cytometry scatter. **A** A schematic illustration of the experimental setup. **B-C** Cell counts of monocytes (B) or lymphocytes (C). *P-value = 0.0229, ***P-value = 0.0002, *P-value = 0.0136 for paired t-tests (from left to right). No significance was observed for the lymphocyte counts.

**
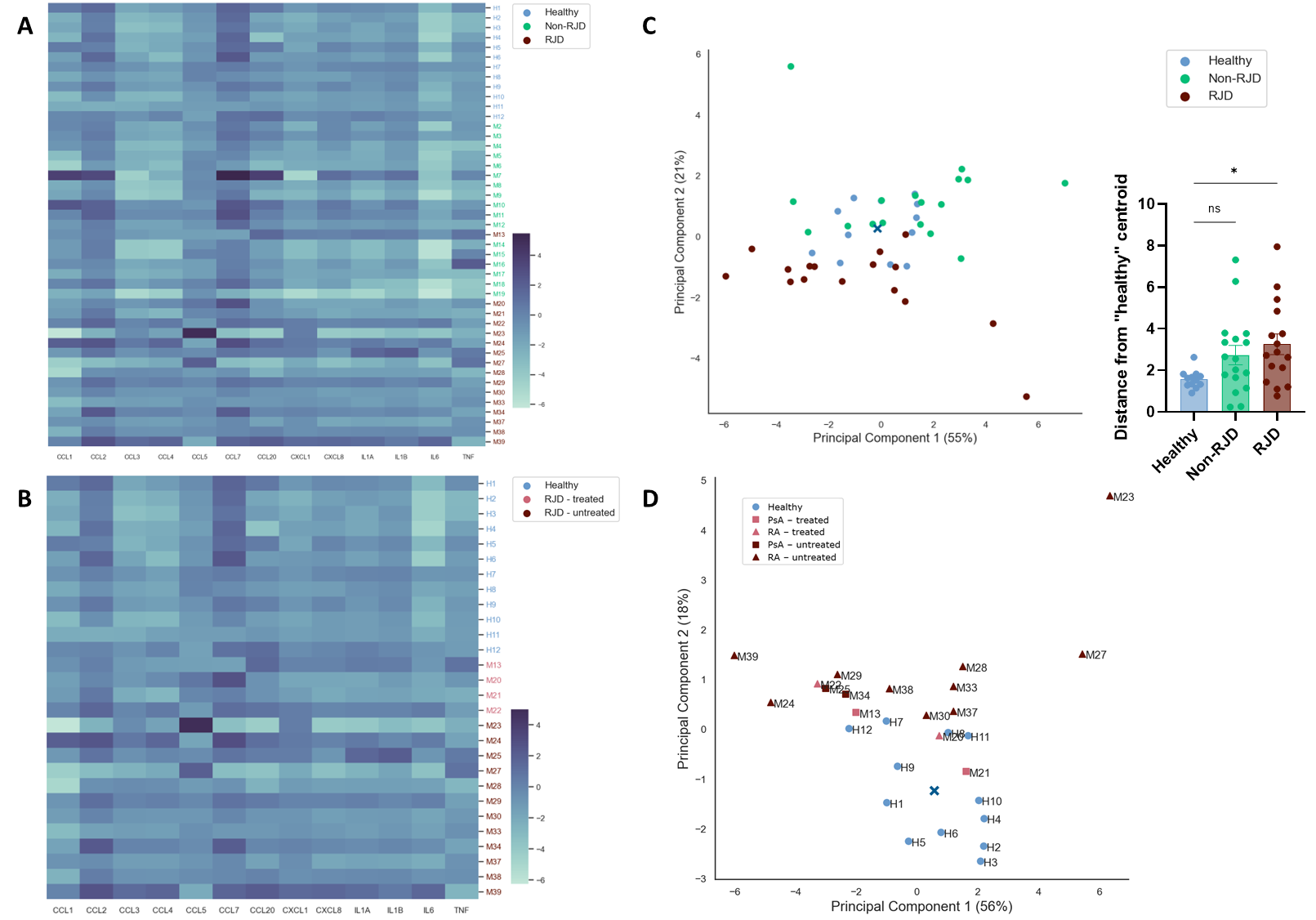
**

**Fig. S6. Data of healthy and patient monocytes treated with Wnt-3a. A-B** Representation of RT-qPCR data of monocytes treated as in Fig. 4E. The continuous color legend represents Log2(Fold Change) of the gene expression of Wnt-3a-treated vs. control-treated monocytes. The tested genes are noted on the x-axis. Please note that data presented in Fig. 4E is used here as “Healthy”. **A** Healthy donors (blue), patients with inflammatory states other than RJD (green) and RJD patients (maroon). These data were used to produce C. **B** Healthy donors (blue), treated RJD patients (pink) and RJD patients naïve to treatment (maroon). These data were used to produce Fig. 7A. **C** Principal component analyses (PCA) based on RT-qPCR data (presented in A) of Wnt-3a-treated monocytes (treated as in Fig. 4E). The blue X represents the centroid of the healthy donor samples, calculated as the average of the x and y coordinates of all the blue dots on the PCA graph. The bar graphs represent a calculation of distance of each dot from the healthy donor centroid, based on x,y coordinates. Healthy donors (blue), patients with inflammatory states other than RJD (green) and RJD patients (maroon). *P-value = 0.0228, ns = 0.1276. **D** PCA plot as in Fig. 7A. Each point is annotated with the corresponding donor number. Point shape represents the donor condition: circle – healthy, triangle – rheumatoid arthritis (RA), square – psoriatic arthritis (PsA). Point color is based on immunosuppressive treatment of RJD patients: pink – treated, maroon and blue– untreated. RJD – rheumatic joint diseases.

**
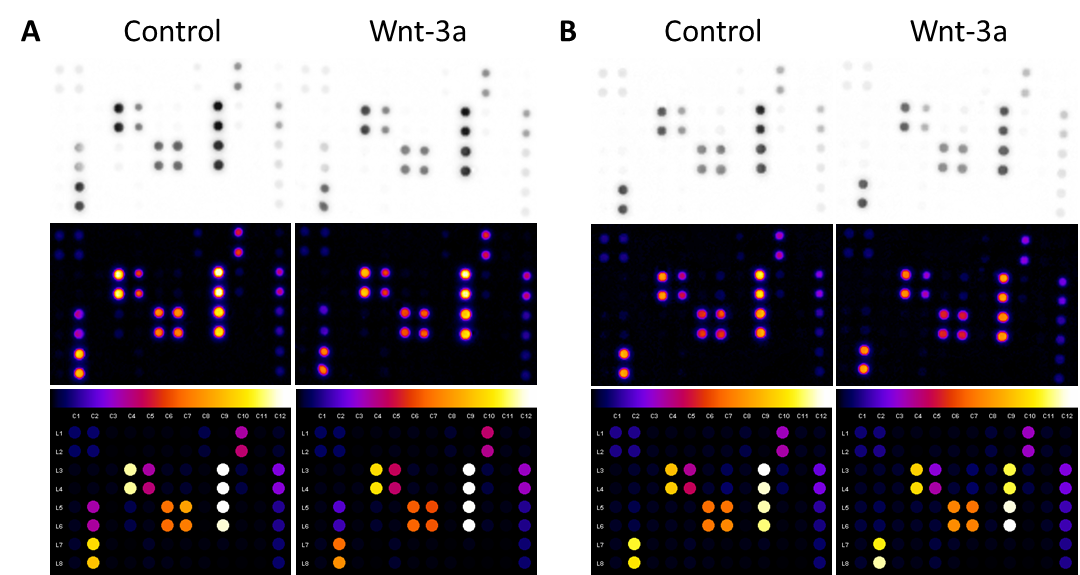
**

**Fig. S7. Quantification of membrane chemokine array (RJD patients).** Media collected from culture of control- and Wnt-3a-treated monocytes were centrifuged twice to remove the cells, subjected to a chemokine membrane array (top panels) and quantified (middle and bottom panels). The experiment was conducted with monocyte from two RJD patients (**A-B**). These data, along with the data presented in Fig. S4, were used to produce Fig. 7C. RJD – rheumatic joint diseases.
